# Prognostic significance of RBP2-H1 variant of JARID1B in melanoma

**DOI:** 10.1186/s12885-017-3836-x

**Published:** 2017-12-15

**Authors:** Łukasz Kuźbicki, Dariusz Lange, Agata Stanek-Widera, Barbara W. Chwirot

**Affiliations:** 1 Department of Medical Biology, Faculty of Biology and Environment Protection, Nicolaus Copernicus University, Lwowska 1, 87-100 Toruń, Poland; 2 Department of Tumor Pathology, Oncology Center – Maria Skłodowska-Curie Institute, Wybrzeże Armii Krajowej 15, 44-101 Gliwice, Poland

**Keywords:** JARID1B, RBP2-H1 variant of JARID1B, Melanoma, Patient’s survival, Immunohistochemistry

## Abstract

**Background:**

Histone demethylase JARID1B plays several context dependent roles in epigenetic regulation of cellular differentiation in normal development and is highly expressed in multiple human cancers. The protein is a strong transcriptional repressor capable of downregulating numerous genes. There are three splicing isoforms of JARID1B, however the links between the protein structure and function are not clear. The expression pattern of JARID1B in human melanoma seems to be different from observed in other cancers. Moreover, up to now no data on the impact of JARID1B expression in cutaneous melanoma on the patients’ prognosis have been reported.

**Methods:**

We investigated immunohistochemically the association of intratumoral expression of total JARID1B protein and its RBP2-H1 isoform in primary and metastatic melanomas with prognosis for the patients.

**Results:**

Expression of both total JARID1B protein and its RBP2-H1 variant was found in all the melanomas investigated. Our results indicate, however, that only high (above 90% of the cells) intratumoral expression of RBP2-H1 can be considered prognostic factor associated with worse overall survival of the patients.

**Conclusions:**

Such results if considered together with data demonstrating a switch to enhanced expression of RBP2-H1 at early stages of malignant transformation of melanocytes are in agreement with hypothetical crucial role of JARID1B in the course of melanoma development and progression and suggest that altered splicing of JARID1B may be important factor increasing melanoma aggressiveness.

**Electronic supplementary material:**

The online version of this article (10.1186/s12885-017-3836-x) contains supplementary material, which is available to authorized users.

## Background

Recent decade brought a large body of evidence indicating significant role of histone H3 lysine 4 (H3K4) JARID1B demethylase in initiation and progression of several human malignancies including prostate, breast, liver, ovary, lung and brain cancers and melanoma [1–12]. Roesch et al. [11, 12] suggested that in melanoma the protein is essential for tumor growth and is a marker of a dynamically changing population of slow-cycling cells required for continuous tumor growth. Isolated JARID1B - positive melanoma cells give rise to rapidly proliferating and heterogeneous progeny and the authors attributed to them a role of putative melanoma initiating cells different in character from cancer stem cells following a hierarchical model. According to Roesch et al. [13] the melanoma cells expressing JARID1B are also responsible for multidrug resistance of melanoma. The authors also suggested that development and progression of melanoma are associated with a general decrease of the JARID1B expression in the tumors compared to benign naevi [11]. However, such a significant drop in the JARID1B expression levels was not confirmed in our immunohistochemical study [14] and in consecutive work of Roesch group [13].

There are three variants of JARID1B generated by alternative splicing: PLU-1, RBBP2H1a and RBP2-H1. The PLU-1 variant consists of 1544aa and the form RBBP2H1a contains additional fragment of 137aa at the N end corresponding to a segment of exon 1 of the gene. The RBP2-H1 isoform contains a sequence of 36aa corresponding to exon 6 localized behind the position of 237aa (absent in PLU-1 and RBBP2H1a) [11, 15]. Specific functions of the isoforms have not yet been elucidated. Alterations of splicing patterns in cancer cells were reported for multiple genes (see for instance [16]). To the best of our knowledge only one study [17] focused on functional analysis of roles played by different isoforms of histone demethylase. The authors studied two isoforms of the KDM2A demethylase in breast cancer cell lines. They further analyzed the Cancer Genome Atlas (TCGA) data on DNA copy number, mutation and overall survival for 976 breast cancer sample and found that the short isoform of KDM2A was more abundant in a subset of the cancers investigated. The final conclusion of that study was that overexpression of the short isoform seemed critical for breast cancer progression. Up to now the expression of JARID1B in cancer cells was studied for specific variants or for a total pool of the protein without analysis of its isoform composition. An interesting result of such studies was a discovery that the PLU-1 variant of JARID1B, normally expressed only in human testes, seems to play a crucial role in the breast cancer [18]. It was shown that overexpression of PLU-1 in breast cancer cells modified expression of 100 genes (81% downregulated) [19].

Our recent work [20] demonstrated for the first time that development of melanoma is associated rather with a change in relative expression levels of individual isoforms of JARID1B and not with enhancement of a total intratumoral expression of the protein. The RBP2-H1 variant was expressed by 75% of the cells of primary melanomas and 85% of the cells of metastatic lesions while in benign naevi its expression could be detected only in 25% of the cells. The intratumoral expression levels of the total JARID1B protein were similar in melanomas and naevi. Taking into account numerous reports on a significance of JARID1B expression for development, progression and drug resistance of multiple human cancers we investigated in this study prognostic significance of the intratumoral expression of the total JARID1B protein and/or its RBP2-H1 variant for patients with skin melanoma.

## Methods

### Patient material

Formalin-fixed paraffin-embedded tissue specimens were obtained from archives of Department of Tumor Pathology at the Maria Skłodowska-Curie Institute, Center of Oncology in Gliwice, Poland. The study consisted of 31 primary melanomas and 17 melanoma lymph node metastases. Primary melanomas were classified according to: Breslow thickness (≤1.0 mm, *n* = 5; >1.0–2.0 mm, *n* = 6; >2.0–4.0 mm, *n* = 8; >4.0 mm, *n* = 12), presence of ulceration (absent, *n* = 9; present, *n* = 22), mitotic index (≤1 mitosis / mm^2^, *n* = 5; >1 mitosis / mm^2^, *n* = 26), Clark infiltration levels (II, *n* = 5; III, *n* = 15; IV, *n* = 6; V, *n* = 5), growth phase (RGP, *n* = 13; VGP, *n* = 18), histologic type (superficially spreading, *n* = 11; nodular, *n* = 19; acral, *n* = 1) and number of lymph nodes with metastases generated by the primary lesions (0, *n* = 13; 1, *n* = 7; 2–3, *n* = 2; ≥4, *n* = 8). Each of the 17 lymph node metastases was paired with corresponding primary melanoma obtained from the same patient. None of 31 patients had received any form of therapy before chirurgical resection of investigated lesions.

### Antibodies

Two different primary antibodies were used: Ab1 - specific only for RBP2-H1 variant of JARID1B and Ab2 - generated against all three isoforms of JARID1B protein (RBP2-H1, RBBP2H1a and PLU-1). Ab1 - mouse monoclonal antibodies, clone 1G10 detect peptide mapped at 231-320aa of the protein (Bio-Rad Laboratories, Oxford, UK). Ab2 - rabbit polyclonal antibodies bind to a sequence (1494-1544aa) at the C-end of the JARID1B protein (Bethyl Laboratories, Montgomery, USA).

### Immunohistochemistry

Expression of JARID1B was detected according to the protocols described in our previous paper [20]. Briefly the 3 μm sections were mounted on 3-aminopropyltriethoxysilane (Sigma-Aldrich, St. Louis, Missouri, USA) coated slides. The sections were dewaxed, rehydrated and incubated in 0.01 M citrate buffer, pH 6.0 at 90°C for 30 min (Ab1) or for 60 min (Ab2) and in 0.3% hydrogen peroxide at room temperature for 30 min to block activity of endogenous peroxidase. Non-specific binding of antibodies was blocked by incubating the sections with 1.5% normal horse (Ab1) or goat (Ab2) serum for 30 min (Vector Laboratories, Burlingame, California, USA). The sections were incubated with primary antibodies either Ab1 (1:25 v/v) or Ab2 (1:5000 v/v) in PBS pH 7.4 either at room temperature for 60 min (Ab1) or at 4°C overnight (Ab2). Immunocomplexes were visualized with respective biotinylated secondary antibodies and ABC technique using the Vectastain Elite ABC Kits (Vector Laboratories) according to the procedures supplied with the kits. The substrate for peroxidase was 0.05% 3,3′-diaminobenzidine (DAB, Sigma-Aldrich) with 0.01% hydrogen peroxide and 0.06% nickel chloride (II) (Sigma-Aldrich) in Tris-HCl buffer, pH 7.4. Negative control sections, processed as in the full protocols but incubated with pure PBS pH 7.4 without primary antibodies did not produce detectable DAB precipitates. Staining of epidermal keratinocytes in several layers of normal skin surrounding primary melanomas constituted positive control.

### Immunohistochemical scoring

Microscopic examinations were conducted using BX61 microscope (Olympus Corporation, Tokyo, Japan) with white light illumination. Digital images were recorded with XC50 camera (Olympus Soft Imaging Solutions, Münster, Germany) and analyzed using Cell^P^ software, version 3.3 (Olympus Soft Imaging Solutions, Hamburg, Germany, 2009). Immunohistochemical scoring was carried out for three fields of view at 1000× magnification and involved counting positively stained melanocytes separately for each of the skin layers in central regions of the lesions.

### Statistical analysis

The final data are presented as box plots with marked median values of percentage fractions of positive cells. The differences in JARID1B expression were evaluated for both Ab1 (*P*_1_) and Ab2 (*P*_2_) using Mann-Whitney *U*-test with the parameter of statistical significance *P*_1,2_ < 0.05. The association of the percentage fraction of the stained cells with patients’ survival time was determined using Kaplan-Meier method and the survival curves were compared using log-rank test. The value of the JARID1B RBP2-H1 isoform expression as an independent prognostic marker was determined using Cox proportional hazards modeling (MedCalc Software, Ostend, Belgium). Potential predictor variables other than RBP2-H1 levels entered into the multivariate analyses included successively expanded groups of clinicopathological characteristics comprising Breslow tumor thickness, ulceration, mitotic index, Clark infiltration level, growth phase, histologic type and number of lymph node metastases.

## Results

**Fig. 1:**
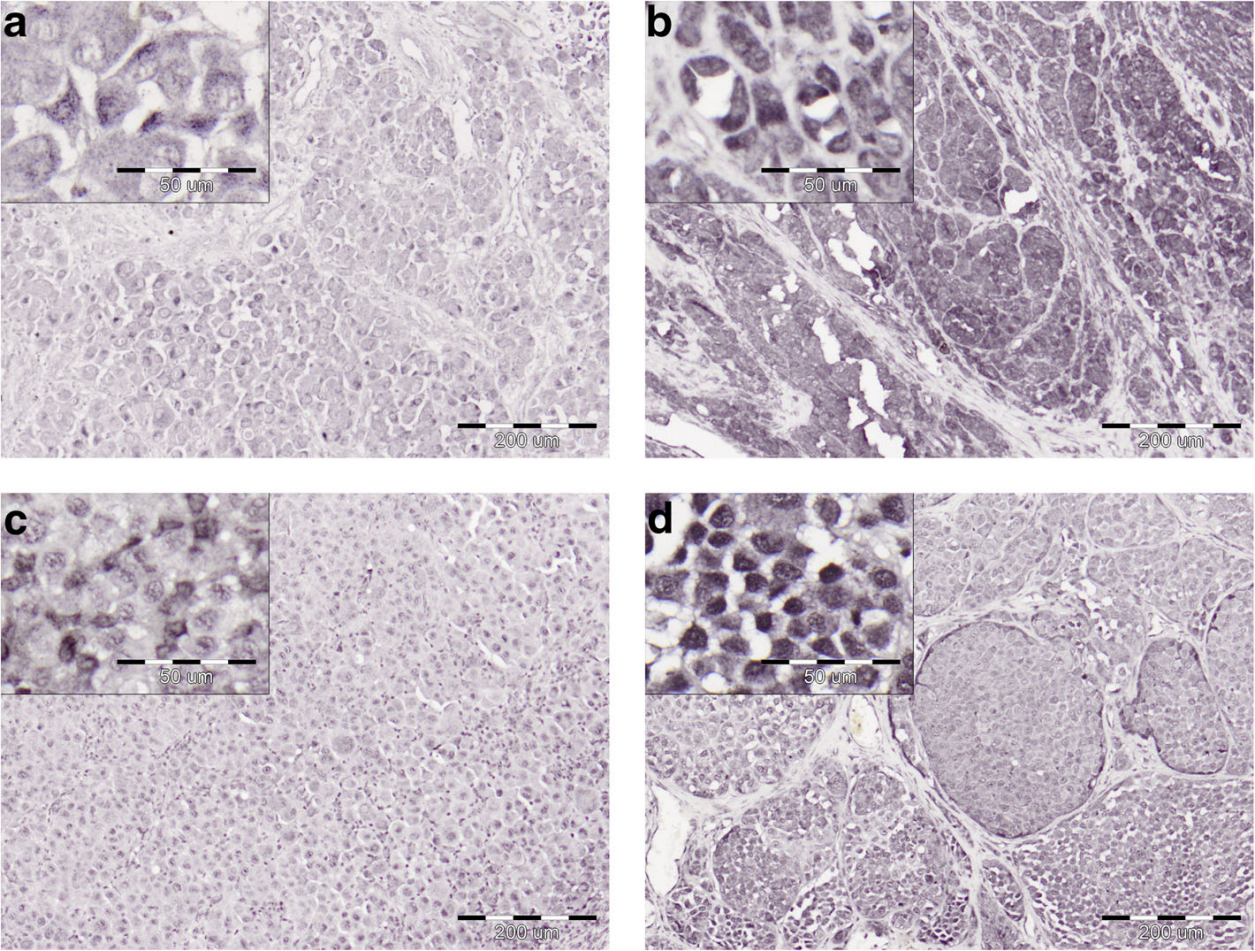
Immunohistochemical staining of JARID1B in primary melanomas. Detection of RBP2-H1 isoform using Ab1 antibody (**a, b**) and total JARID1B protein using Ab2 antibody (**c, d**) in: nodular melanoma (T3bN0Mx, patient’s survival: 156 months) (**a, c**) and superficially spreading melanoma (T3bN3M1c, patient’s survival: 17 months) (**b, d**). Central regions of the lesions, original magnification 100×, insets 400×

**Fig. 2:**
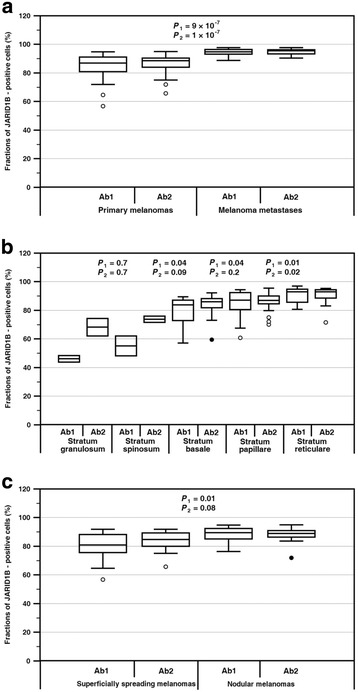
Quantitative analysis of expression of RBP2-H1 variant and total JARID1B protein. Percentage fractions of JARID1B – positive cells in primary melanomas and melanoma lymph node metastases (**a**), in different skin layers of the primary melanomas (**b**) and in primary melanomas of different histologic types (**c**). The *P*_1_ and *P*_2_ parameters concern the results obtained with the Ab1 and Ab2 antibodies, respectively (Mann-Whitney *U*-test)

Expression of both total JARID1B and its RBP2-H1 variant was observed in all the primary and metastatic melanomas investigated. Raw data on JARID1B expression and patients’ overall survival including the main tumor characteristics are given in Additional Supporting Files. Representative images of immunohistochemical staining are presented on Fig. 1. Although JARID1B as a functional histone demethylase should be localized to nuclei the staining was either nuclear or both nuclear and cytoplasmic. Similar staining patterns were, however, observed also in earlier studies [11, 12, 18, 20]. The mean percentage fractions of the cells stained for RBP2-H1 were only slightly lower than the fractions of the cells with the staining positive for the expression of all the three isoforms of JARID1B (Fig. 2a). Such results suggest dominating contribution of the RBP2-H1 to intratumoral expression of the JARID1B protein and fully agree with our earlier observations [20].

Lymph node metastases expressed both the proteins at higher levels than the primary lesions (Fig. 2a). If the correlated pairs of primary and metastatic melanomas were analyzed, the expression of the RBP2-H1 variant was consistently higher in metastatic lesions in all the 17 cases examined. In the case of the total JARID1B, two metastatic lesions expressed the protein at levels equal or lower than their primary counterparts. In the primary melanomas the percentage fractions of the labeled cells changed with the distance from the skin surface (Fig. 2b) increasing by a factor of two from the granular to the reticular layer. The observed gradient was statistically more significant for the RBP2-H1 isoform.

**Fig. 3:**
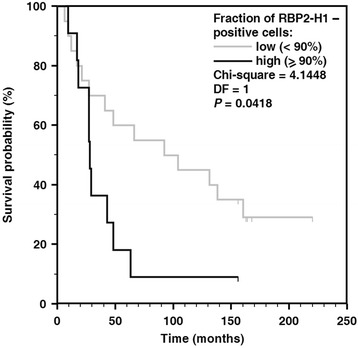
Kaplan-Meier analysis of patients’ overall survival and expression of RBP2-H1 protein in primary melanomas. The expression levels were classified as low or high according to 90% cut-off threshold of the percentage fractions of immunohistochemically stained cells

No statistically significant correlation was found between the expression levels of the total JARID1B protein and patients’ survival. Such a correlation was found, however, for the RBP2-H1 isoform for the threshold level of 90% of the cells of primary lesions expressing this protein (Fig. 3 and Table 1). The enhanced RBP2-H1 expression in primary melanomas was associated with hazard ratio amounting to 2.18 (95% confidence interval: 1.04 to 7.36). Results of Kaplan-Meier analysis of patients’ overall survival and expression of RBP2-H1 protein in primary melanomas at cut-off thresholds of the percentage fractions of immunohistochemically stained cells of 70% 75%, 80% and 85% are given in Additional Supporting Files.

**Table 1:** Expression of the RBP2-H1 variant of JARID1B protein in primary melanomas and patients’ overall survival

	Survival time (months)	
Percentage fraction of the stained cells	Mean	Median
Low (<90%) *n* = 20	95	98
High (≥90%) n = 11	42	28
Overall *n* = 31	76	48

No correlations were also found between the expression of the proteins of interest and histologic and clinical prognostic characteristics comprising Breslow thickness, Clark levels, growth phase, mitotic index, ulceration of the lesions or number of lymph node metastases (Mann-Whitney *U*-test). The expression levels of both the total JARID1B protein the RBP2-H1 isoform were, however, significantly higher in nodular melanomas compared to the superficially spreading ones (Fig. 2c).

The multivariate analysis of the RBP2-H1 expression and clinicopathological features of melanoma yielded statistically significant results. Such an approach based on Cox proportional hazards modeling showed the association between high level of RBP2-H1 – positive melanoma cells and worse patients’ overall survival (Table 2). The low *P* values indicate that the RBP2-H1 expression is a melanoma prognostic marker independent of the clinicopathological features included in the analysis.

**Table 2:**
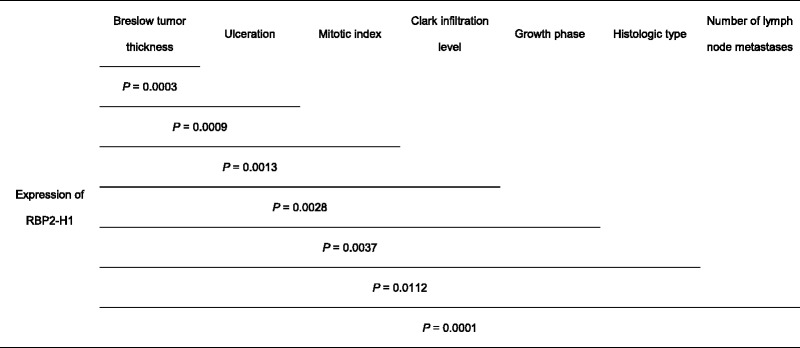
Evaluation of the RBP2-H1 expression in primary lesions as an independent prognostic marker for melanoma

Statistical significance of correlations between patients’ overall survival and percentage fractions of RBP2-H1 – positive cells assessed using Cox proportional hazards modeling including groups of clinicopathological tumor characteristics

## Discussion

The associations between JARID1B and development and progression of melanoma as well as prognosis have not been yet elucidated. Roesch et al. [12] suggested that expression of the protein is a characteristic feature of the subpopulation of cells crucial for a continuous growth of the tumors. According to the authors, among different subpopulations of heterogeneous melanoma tumors there is a subpopulation of slow-cycling cells overexpressing JARID1B. The expression of JARID1B is dynamically regulated and the JARID1B expressing cells are supposed to play a role of quasi-stem melanoma cells important both for the progression of the disease and for drug resistance of melanomas. However, Roesch et al. [11–13] studies were carried out mostly using melanoma cell lines. Immunohistochemical studies involving relatively large sample of human melanomas [14] demonstrated that both primary and metastatic melanomas expressed higher levels of JARID1B compared to benign naevi. In our recent study [20] the expression of JARID1B was detected using two types of primary antibodies specific either to the total JARID1B protein or only to its RBP2-H1 isoform. The results indicated that malignant transformation of melanocytes is associated not so much with enhancement of the total JARID1B expression but rather with a change of the expression pattern of its isoforms with a significant increase of intratumoral expression of the RBP2-H1 variant in melanomas compared to naevi. Significance of relative abundance of different isoforms of the KDM2A histone demethylase in cancer was recently demonstrated by Liu et al. [17] who found that amplification and resulting overexpression of the short isoform of the protein is significant for a progression of at least a subset of breast cancers. The present data also demonstrate a significance of the enhanced RBP2-H1 expression for development and progression of human melanoma. Our results show a specific pattern of changes of the RBP2-H1 expression in primary melanomas depending on a location of the cells within skin layers. The highest expression was observed for the cells infiltrating deeper skin layers and the correlation of the expression levels with a distance from the skin surface was statistically significant. Similar changes could be observed for the expression of the total JARID1B protein but in that case the correlation was not significant. A significance of the RBP2-H1 for melanoma progression is further reflected in the enhanced expression of the protein in metastatic lesion compared to primary melanomas. Such an enhancement was found for all the correlated pairs of primary and metastatic lesions.

Intratumoral expression of RBP2-H1 was also stronger in nodular melanomas compared to less malignant superficially spreading melanomas. No correlations were found between the expression levels of the both protein classes investigated and a progression of the disease (assessed according to Breslow thickness or Clark levels and growth phases) or other clinicopathological characteristics such as ulceration, mitotic index of the lesions or number of lymph node metastases.

It seems that the enhancement of the intratumoral expression of RBP2-H1 isoform of JARID1B is an early event in malignant transformation of melanocytes and is required for continuous development and progression of melanoma at every stage of the disease. If so it is not surprising that the expression of both the total JARID1B protein and its RBP2-H1 variant are not strongly associated with the survival of the patients. Statistically significant worsening of the prognosis was found only for melanomas with at least 90% of the cells expressing RBP2-H1.

The main goal of the present study was to examine the impact of JARID1B expression in cutaneous melanoma on the patients’ prognosis. It is obvious that the present investigation does not allow for a clear mechanistic explanation of the results. The worsening of the prognosis is clearly observed only at high intratumoral expression of the RBP2-H1 isoform. Such observation indicates that the change of the expression pattern of the JARID1B isoforms modifies but only to limited extend cellular functions of this demethylase resulting in a slight but significant increase of aggressiveness of the cells already at a stage of primary lesions. The presence of the additional exon 6 modifies conformation of the protein and influences its substrate specificity. Taking into account a wide spectrum of genes potentially influenced by JARID1B its difficult or even impossible to point those responsible for the discovered worsening of the prognosis of some melanoma patients. Nevertheless, it seems interesting to notice that JARID1B is one of proteins interacting with Ser/Thr protein phosphatases (PP1 and PP2A) [21]. The binding to phosphatases is mediated by docking motifs and is sensitive to conformation of the interacting proteins and may lead to both inhibition and promotion of enzymatic activity. Li et al. [22] found that inhibition of PP1 and PP2A lead to higher activity of Akt with resulting increase of survival of prostate cancer cells while Facompre et al. [23] proposed that increased activity of Akt increases JARID1B levels which initiates further Akt activation by repressing PTEN and enhancing by this mechanism PI3K/Akt signaling. Assuming that binding of RBP2-H1 isoform inhibits to some extent activity of PP1 and PP2A one might hypothesize that the effect could lead to enhancement of the processes discussed by Facompre et al. [23].

## Conclusions

High level of intratumoral expression of the RBP2-H1 protein is likely to be associated with accelerated progression of the disease resulting in shortening of the survival time of the patients.

## Additional files

Additional file 1: Raw data on JARID1B expression and patients’ overall survival including the main tumor characteristics. (XLS 26 kb)

Additional file 2: Kaplan-Meier analysis of patients’ overall survival and expression of RBP2-H1 protein in primary melanomas. The expression levels were classified as low or high according to following cut-off thresholds of the percentage fractions of immunohistochemically stained cells: 70% (a), 75% (b), 80% (c) and 85% (d). (TIFF 2994 kb)
