# Supplementary figures and images for: Prognostic significance of RBP2-H1 variant of JARID1B in melanoma
Source: BMC Cancer. 2017 Dec 15;17:854. doi: 10.1186/s12885-017-3836-x (PMC5731204; doi:10.1186/s12885-017-3836-x)

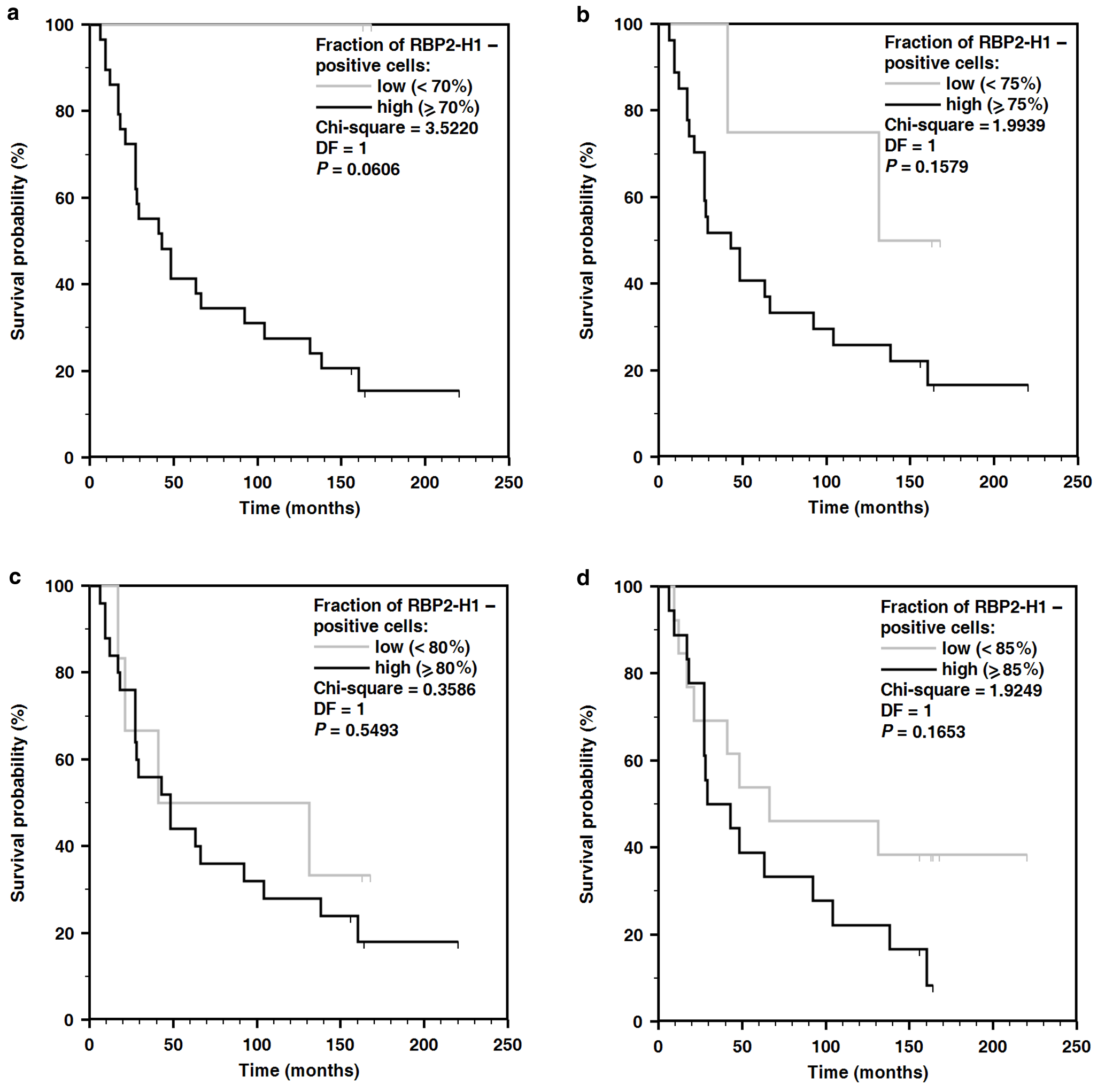

Supplement: Supplementary file 2 — Kaplan-Meier analysis of patients’ overall survival and expression of RBP2-H1 protein in primary melanomas. The expression levels were classified as low or high according to following cut-off thresholds of the percentage fractions of immunohistochemically stained cells: 70% (a), 75% (b), 80% (c) and 85% (d). (TIFF 2994 kb) [file 12885_2017_3836_MOESM2_ESM.tif]
